# Assembly of CdS Quantum Dots onto Hierarchical TiO_2_ Structure for Quantum Dots Sensitized Solar Cell Applications

**DOI:** 10.3390/ma8052376

**Published:** 2015-05-05

**Authors:** Syed Mansoor Ali, Mohamed Aslam, W. A. Farooq, Amanullah Fatehmulla, M. Atif

**Affiliations:** 1 Department of Physics and Astronomy, College of Sciences, P.O. Box 2455, King Saud University, Riyadh 11451, Saudi Arabia; E-Mails: muhd.aslam@gmail.com (M.A.); awazirzada@ksu.edu.sa (W.A.F.); aman@ksu.edu.sa (A.F.); 2 National Institute of Laser and Optronics, Nilore, Islamabad 45650, Pakistan

**Keywords:** hierarchical TiO_2_ structure, nanourchins, CdS quantum dots, FETEM, FESEM, UV-Vis spectroscopy

## Abstract

Quantum dot (QD) sensitized solar cells based on Hierarchical TiO_2_ structure (HTS) consisting of spherical nano-urchins on transparent conductive fluorine doped tin oxide glass substrate is fabricated. The hierarchical TiO_2_ structure consisting of spherical nano-urchins on transparent conductive fluorine doped tin oxide glass substrate synthesized by hydrothermal route. The CdS quantum dots were grown by the successive ionic layer adsorption and reaction deposition method. The quantum dot sensitized solar cell based on the hierarchical TiO_2_ structure shows a current density *J_SC_ =* 1.44 mA, *V_OC_ =* 0.46 V, *FF =* 0.42 and *η* = 0.27%*.* The QD provide a high surface area and nano-urchins offer a highway for fast charge collection and multiple scattering centers within the photoelectrode.

## 1. Introduction

In the modern age, solar cells have attracted significant attention due to their promising applications in energy generation devices. Since the pioneering report by O’Regan and Grätzel in 1991, dye-sensitized solar cells have been investigated extensively all over the world [1,2,3,4,5,6,7,8,9,10,11]. The quantum dot sensitized solar cell (QDSSC) has received wide attentions recently because they have several advantages over dye sensitizers, such as tunable energy gaps [12], high absorption coefficients [13], and generation of multiple electron-hole pair with high energy excitation [14].

The TiO_2_ nanoparticle based photoelectrode showed considerable power conversion efficiency over a large surface area with the attachment of dye molecules. M. Pavan *et al.* reported on an oxide heterojunction solar cell, entirely produced by spray pyrolysis onto fluorine doped tin oxide (FTO) covered glass substrates [15]. However, the irregular stacking of TiO_2_ nanocrystallines have been found to limit the electron transportation and decreases the electron life time because of the random network of crystallographically misaligned crystallites, and lattice mismatches at the grain boundaries [15,16,17,18]. It has been accepted that the value of power conversion efficiency of photoelectrodes highly depends on the morphology and structure of TiO_2_. In order to increase the photovoltaic performance, through their excellent electron transport and light scattering ability, one-dimensional nanostructures, such as nanorods (NRs), nanowires (NWs) or nanotubes (NTs), have been studied as photoelectrode materials for sensitized solar cells [19,20,21,22,23]. Due to low specific surface area ascribing to larger diameter and wide gaps among neighbor NWs [23,24], the TiO_2_ NWs based photoelectrode has not shown remarkable enhancement of power conversion efficiency. To overcome this problem, hierarchically-structured materials composed of nanocrystallites that form large micro-spheres. Nanocrystallites can provide excellent light scattering with large surface area for sensitizer-uptake. In these hierarchical materials, slow trap-limited charge transport remains a fundamental problem. To solve this problem, a nanourchin (NU) TiO_2_ are formed by clustering nanowires that have a mean diameter of about 50 nm and a length of a few micrometers to construct a radially aligned structure. There are few recent examples concerning hierarchical TiO_2_ structure (HTS), such as either for rutile TiO_2_ on FTO glass or anatase TiO_2_ on a Ti foil substrate for improving the power conversion efficiency [19,24].

In the present work, hierarchical TiO_2_ structure (HTS) consisting of spherical nano-urchins was synthesized through hydrothermal method. The CdS QDs were assembled by successive ion layer adsorption and reaction (SILAR). The HTS/CdS QDs based photoelectrode was used to improve the power conversion efficiency of quantum dot sensitized solar cell.

## 2. Experimental

### 2.1. Synthesis of Hierarchical TiO_2_ Structure

The hierarchical TiO_2_ structure (HTS) was grown on the FTO substrate. In a typical synthesis, the substrate was ultrasonically cleaned sequentially in acetone, isopropyl alcohol and deionized water for 15 min and was finally dried with nitrogen flow. Separately, 1 mL of titanium isopropoxide was added dropwise to a 1:1 mixture of deionized water and concentrated (35%) hydrochloric acid to obtain a clear transparent solution. The substrate was placed at an angle in a 100 mL Teflon liner, and the precursor solution was added to it. The Teflon liner was loaded in an autoclave and was placed in furnace. The growth was carried out at 180 °C for 15 h.

### 2.2. Deposition of CdS Quantum Dots (QD)

The CdS quantum dots were deposited on HTS films by successive ionic layer deposition and reaction (SILAR) method. The HTS electrode was exposed to Cd^2+^ and S^2−^ ion successive immersion in a ethanolic solutions of 0.5 M Cd(NO_3_)_2_ and methnolic solution of 0.5 M Na_2_S. The film was dipped into 0.5 M Cd(NO_3_)_2_ solution for 1 min and rinsed with ethanol and then, dipped into 0.5 M Na_2_S for 1 min and rinsed with methanol. These dipping procedures are considered one cycle. The coating procedure was repeated 10 times. For the deposition of CdS on the HTS by SILAR method, the experimental procedure is explained as follow:

$$(\mathrm{HTS})\overset{\;{\text{Cd}}^{\text{2+}}\;}{\to }(\mathrm{HTS})\text{C}{\text{d}}^{2+}\overset{\;}{\to }\text{Rinsed}\overset{\;{\text{S}}^{2-}\;}{\to }(\mathrm{HTS})\mathrm{Cds}\overset{\;}{\to }\text{Rinsed}$$

### 2.3. Preparation of Electrolyte Solution

Polysulfide electrolytes were prepared by mixing suitable quantities of Na_2_S, S, and KCl powders in water/methanol solution taken in the ratio 3/7.

### 2.4. Fabrication of QDSCs

**Figure 1:**
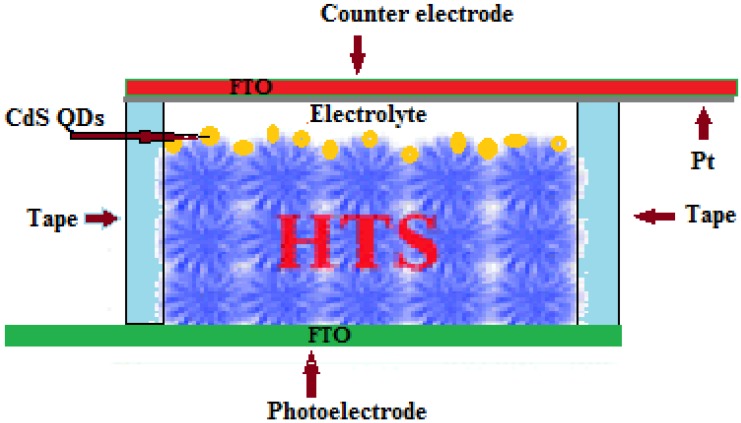
Schematic of the Hierarchical TiO_2_ structure (HTS) based quantum dot sensitized solar cell (QDSSC).

The QD-adsorbed HTS was used as the working electrode and platinum coated FTO glass as counter electrode. The electrodes were assembled into a sealed cell with a cello tape spacer and binder clips, with an active area equal to 0.36 cm^2^. The electrolyte was injected from the edges into the open cell, and the cell was tested. The schematic of the studied QDSSCs is shown in Figure 1.

### 2.5. Characterization

X-Ray diffraction XRD analysis of HTS/FTO films was carried out using multipurpose X-ray diffractometer (Bruker D8 Discover, Bruker AXS GmbH, Karlsruhe, Germany) with Cukα source radiation. Surface morphology of the films was investigated with a JEOL (JSM-7600F) Field Emission electron microscope (Jeol, Peabody, MA, USA). Size of the CdS QDs was measured by JEOL (JEM-2100F) field emission electron microscope (FETEM) (Jeol, Peabody, MA, USA). Optical absorption studies were made at room temperature by using UV-Vis-NIR spectrophotometer (JASCO-V 670) (Jasco, Halifax, NS, Canada) in the wavelength range 200 nm–800 nm. The current-voltage and capacitance-voltage characteristics were investigated using a Semiconductor Characterization System SC-4200 from Keithley (Keithley Instruments, Solon, OH, USA). The films were illuminated by a Class-BBA Solar Simulator (PV measurements, Boulder, CO, USA) and TM-206 solar power meter (Tenmars, Taipei, Taiwan) was used for measuring the light intensity.

## 3. Results and Discussion

**Figure 2:**
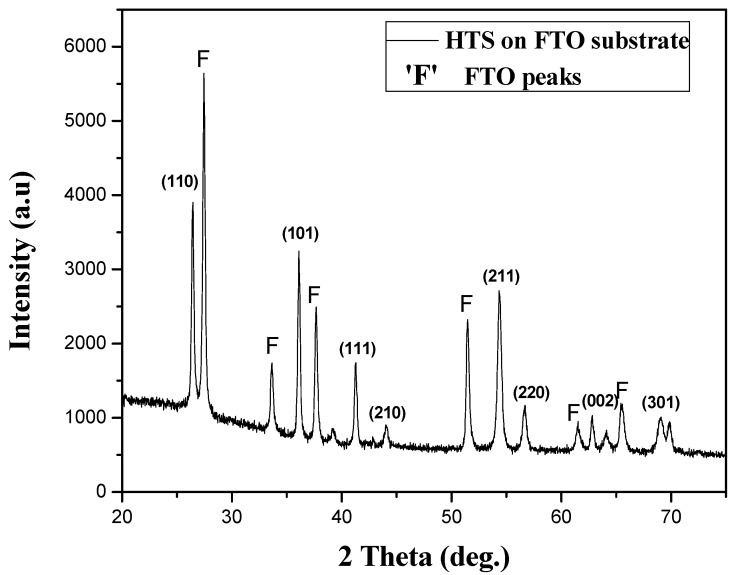
X-ray diffraction pattern of hierarchical TiO_2_ structure on fluorine doped tin oxide (FTO) glass substrate.

Figure 2 shows the XRD patterns of TiO_2_ nanowires grown on FTO glass substrate. Nanowires grown in this work, regardless of substrate used, were found to have the rutile phase by matching between the observed and standard “*d*” value of the TiO_2_ nanostructure. XRD data (Figure 1) show a good agreement with the standard TiO_2_ (PDF file #01-086-0147, P4_2_/mnm, a = b = 4.594 Å and c = 2.958 Å). In XRD spectrum, the respective diffraction peaks corresponding to the FTO are denoted by the symbol “F”.

**Figure 3:**
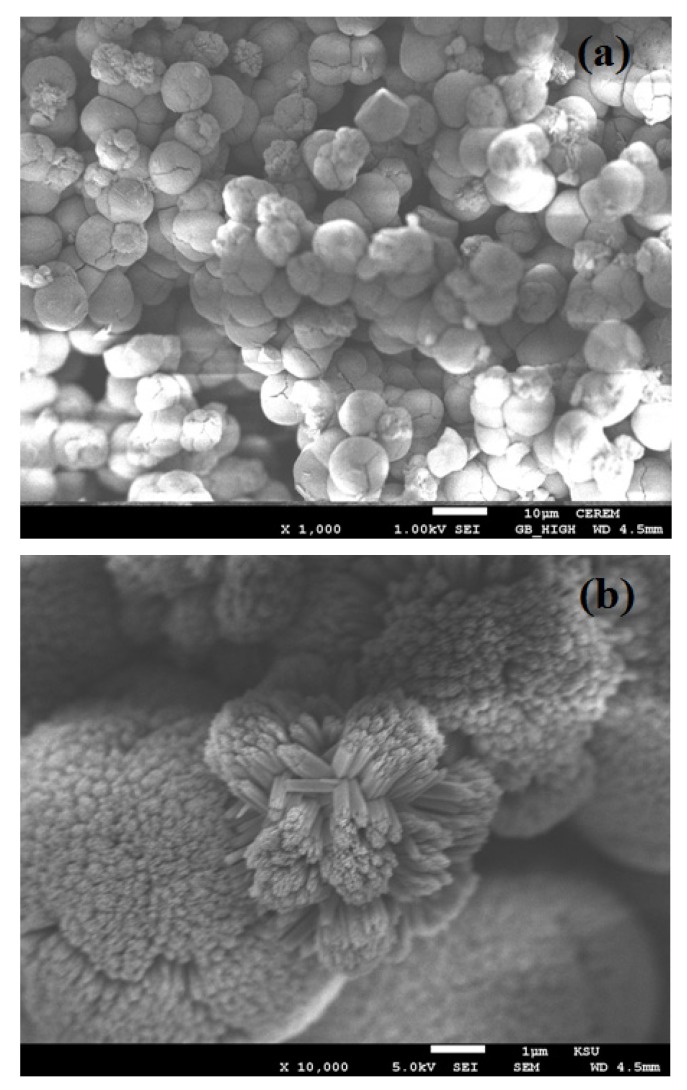
Field emission scanning electron microscopy images of hierarchical TiO_2_ structure. (**a**) at low resolution (1000 X); (**b**) at high resolution (10,000 X).

The field emission scanning electron microscope (FESEM) images of TiO_2_ nanostructure on FTO glass is shown in Figure 3. From Figure 3a,b we have found that the morphology of TiO_2_ is a hierarchical structure. It is believed that a hierarchical TiO_2_ structure (HTS) has three novel levels. The first level, the TiO_2_, is made up of nano-urchins (NUs); the second level, the NUs, is composed of nanowires (NWs); and the third level, the NWs, is made up of nanoparticles (NPs) [25]. Figure 3a depicts the NUs grown on the FTO glass substrate with an average diameter of 8 µm. Higher magnification of FESEM image Figure 3b reveals the TiO_2_ nano-urchins is composed of the TiO_2_ nanowires of average length of 1 µm.

**Figure 4:**
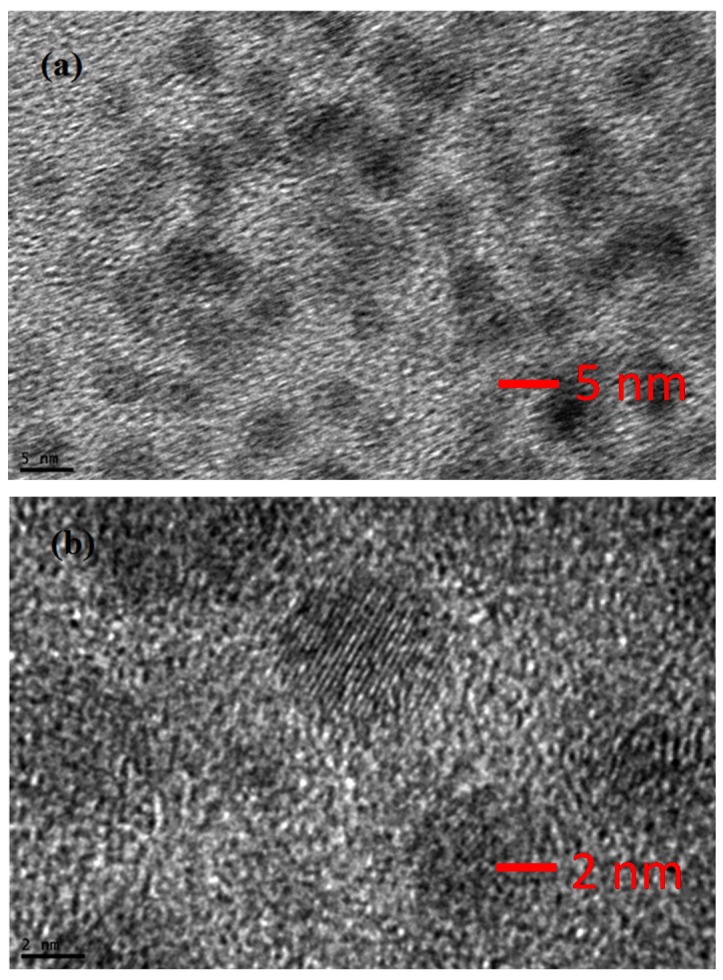
Field emission transmission electron microscopy images of CdS QDs in (**a**) low resolution and 5 nm (**b**) High resolution 2 nm.

Figure 4a,b shows the low and high magnification TEM images of CdS QDs nanoparticles deposited by SILAR technique on the TiO_2_ nanostructure. A clear morphology of CdS QDs nanoparticles, with a size from 4 nm to 6 nm, indicates that CdS QDs are markedly immersed on the surface of TiO_2_ nanostructure.

**Figure 5:**
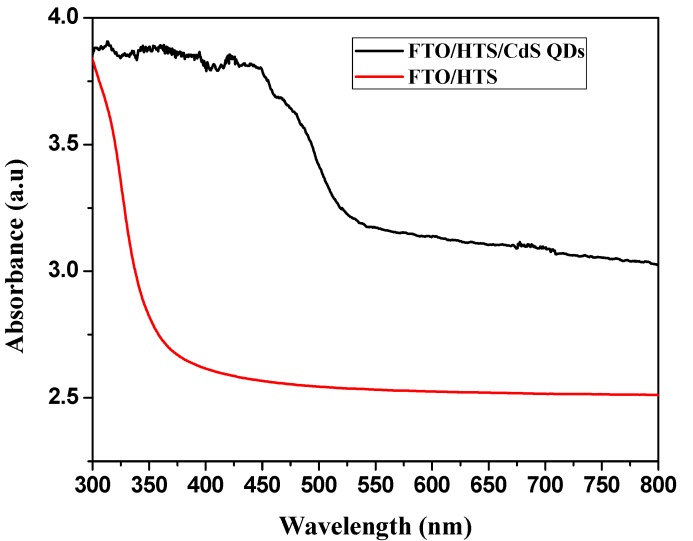
Absorbance spectra of HTS and HTS/CdS QDs.

The comparison of the absorption spectra of HTS and CdS QDs deposited on the HTS is shown in Figure 5. The immersion of CdS QDs in HTS structure has improved the optical absorbance in the visible region. The absorption edge obtained from the intersection of the sharply decreasing region of a spectrum with its baseline for HTS around 370 nm and shifted to longer wavelength around 520 nm after immersion of CdS QD. Corresponding to this absorption peak, the band gap was calculated to be 2.7 eV. The value reported for CdS in bulk was 2.42 eV [26]. The band gap of CdS particles deposited on HTS films was higher than that of CdS bulk, which indicated that the size of the CdS particles were still within the scale of quantum dot. Estimated from the absorption edge of the absorption spectra, the radius of CdS particles was calculated to be 2.37 nm by using the hyperbolic band model (HBM) equation [27].

(1) $$R=\sqrt{\frac{h^{2}E_{bulk}}{2m^{*}\left(E_{nano}^{2}-E_{bulk}^{2}\right)}}$$

where *E_bulk_* is bulk band gap; *E_nano_* is band gap of nanomaterial; m* is effective mass of electron in bulk CdS (m* = 0.21m_o_). Hence the particle size estimated as 2*R* is 4.74 nm.

A new approach implemented the benefits of one-dimensional nanostructures by suitably combining the NU TiO_2_ and nanoparticles (NP) to construct a hierarchical TiO_2_ structure (HTS) photoelectrode for the QDSSCs. In particular, the QD provide a high surface area for sufficient QD deposition by SILAR technique, whereas NU TiO_2_ particles offer a highway for fast charge collection and multiple scattering centers within the photoelectrode. The QDSSCs made of the HTS film exhibited remarkable improvement in power conversion efficiency in comparison to the reference cell made with the NP film [28,29,30].

**Figure 6:**
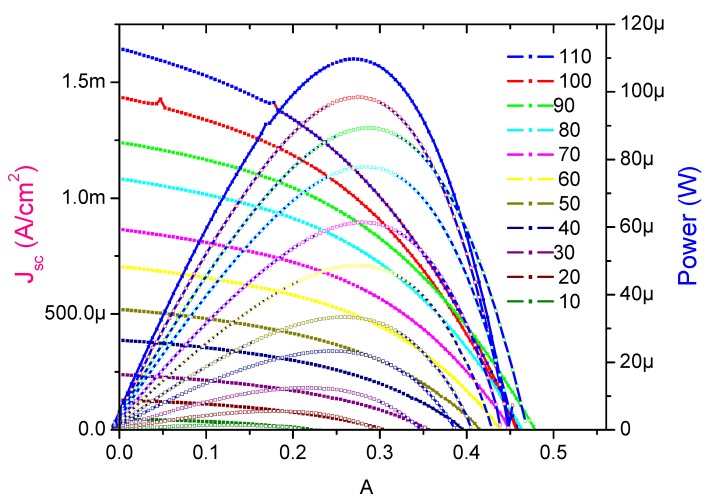
*J–V* and *P–V* characterization of HTS based QDSSC at different intensities of light.

To evaluate the photovoltaic performance of the HTS, the synthesized products were applied as a photoanode for QDSSC application. The *J–V* characteristics of the quantum dot sensitized (FTO/HTS/CdS QDs/Pt/FTO) solar cell were measured using a solar simulator. The fourth quadrant of *J–V* and *P–V* characteristics are shown in Figure 6. The photocurrent is defined as a current produced under light irradiation due to the generation of free charge carriers by absorption of photons within the depletion layer. Figure 6 demonstrate that the value of *J* increases with the increasing illumination intensities and the photovoltage value increased up to 0.478 V at 90 mW/cm^2^ then decreases to 0.46 V at100 mW/cm^2^. The values of short circuit current density *J_sc_* and open circuit voltage *V_oc_* are found to be 1.44 mA/cm^2^ and 0.46 V under 100 mW/cm^2^ illuminations, respectively, with an efficiency of 0.27%. As per the literature, we expected the assembly of CdS quantum dots onto hierarchical TiO_2_ structure for quantum dots sensitized solar cell to give good results. However, with our fabricated cell, we do not observe similar results. The probable reason for obtaining lower efficiency may be due to the QDs embedded in the TiO_2_ nano urchins matrix provides many pathways, resulting in an increase in injection time.

By power-voltage curve, we can take more information about the delivered power to this device. The electric power first increases with the increasing of the voltage and reaches its maximum value and then, decreases and reaches to zero with the further increase of the voltage. The maximum value of power indicates how much the QDSSC can deliver as a maximum power to an external load and is defined as *P_max_* = *I_M_ × V_M_*, where *I_M_* is the maximum current and *V_M_* is the maximum voltage at each value of illumination intensities.

**Figure 7:**
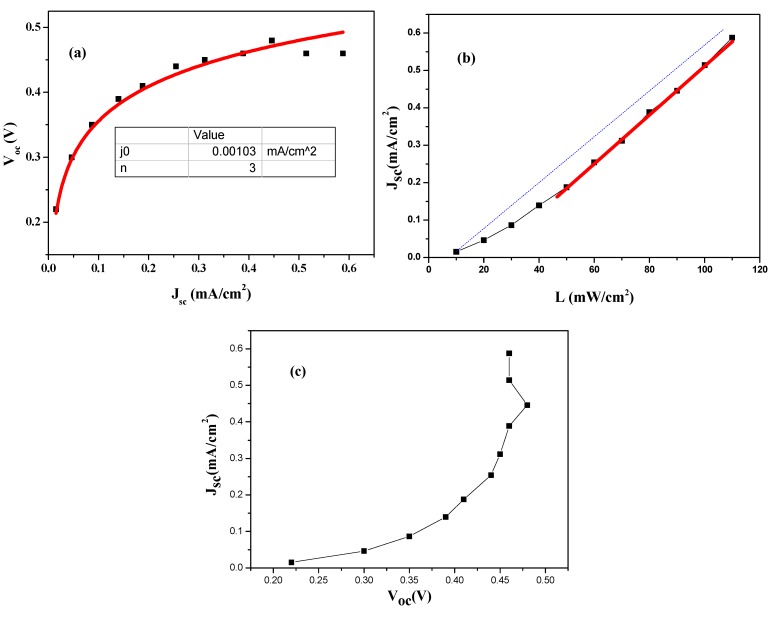
Photovoltaic performance parameter of HTS based QDSSC (**a**) *V_oc_−J_sc_*; (**b**) *J_sc_*–*L*; (**c**) *J_sc_*−*V_oc_* plot.

Figure 7a shows the graph between the short circuit current density *J_sc_* with open circuit voltage *V_oc_*. It is seen that the value of *V_oc_* increases exponentially with the increase of *I_sc_*. This exponentially increasing trend of *V_oc_* with *I_sc_* obeys the following relation [31,32].

(2) $$V_{oc}=\frac{nkT}{q}ln\left(\frac{J_{sc}}{J_{o}}+1\right)$$

where *n* is the diode ideality factor; *k* is the Boltzmann’s constant; *q* is the electric charge; and *J_o_* is the reverse saturation current density. By using the above equation, fitted to the graph of *V_oc_−J_sc_*, the ideality factor of fabricated cell is found to be 3 [33]. For an ideal *p-n* junction, the ideality factor is considered to be approximately 1 under room temperature. The probable reasons for larger ideality factors may be due to several defects as well as local non-linear shunts anywhere in the cell area that are responsible the ideality factor value greater than 1 [34].

The value of short circuit current density *J_sc_* of the solar cell were obtained from *J–V* curves under light illumination. In Figure 7b it is observed that there exists a non-linear relation between current and light intensity in the low intensity region. This is due to the large shunt resistance in the device. The linear relation is fully recovered after the light intensity is increased to 40 mW/cm^2^, indicating that the photo filling effect has saturated the non-radiative recombination center.

Figure 7c show the *J_sc_−V_oc_* relation plot under different light intensity and reveals that the dark diode property of the fabricated with high series resistance.

## 4. Conclusions

A novel hierarchical TiO_2_ structure on transparent conductive FTO glass substrate is synthesized by hydrothermal route. The CdS quantum dots were grown by the successive ionic layer adsorption and reaction deposition method. The investigations of FESEM reveal that HTS consist of spherical nano-urchins and FETEM indicate that CdS QDs are markedly immersed on the surface of TiO_2_ nanostructure. The QD provide a high surface area and nano-urchins offer a highway for fast charge collection and multiple scattering centers within the photoelectrode, which are responsible for the improvement of power conversion efficiency. We anticipate that nano-urchins consisting of HTS based photoelectrodes could be promising for the fabrication of high efficiency Perovskite quantum dot sensitized solar cell.
